# Generation and Applications of the Hydroxide Trihydrate Anion, [OH(OH_2_)_3_]^−^, Stabilized by a Weakly Coordinating Cation

**DOI:** 10.1002/anie.201908589

**Published:** 2019-09-09

**Authors:** Robin F. Weitkamp, Beate Neumann, Hans‐Georg Stammler, Berthold Hoge

**Affiliations:** ^1^ Centrum für Molekulare Materialien Fakultät für Chemie Universität Bielefeld Universitätsstraße 25 33615 Bielefeld Germany

**Keywords:** hydroxide hydrate, phosphazene base, Ruppert–Prakash reagent, trifluoromethylation, weakly coordinating cations

## Abstract

The reaction of a strongly basic phosphazene (Schwesinger base) with water afforded the corresponding metastable hydroxide trihydrate [OH(OH_2_)_3_]^−^ salt. This is the first hydroxide solvate that is not in contact with a cation and furthermore one of rare known water‐stabilized hydroxide anions. Thermolysis in vacuum results in the decomposition of the hydroxide salt and quantitative liberation of the free phosphazene base. This approach was used to synthesize the Schwesinger base from its hydrochloride salt after anion exchange in excellent yields of over 97 %. This deprotonation method can also be used for the phosphazene‐base‐catalyzed preparation of the Ruppert–Prakash reagent Me_3_SiCF_3_ using fluoroform (HCF_3_) as the trifluoromethyl building block and sodium hydroxide as the formal deprotonation agent.

## Graphical abstract

**No contact with the cation**: The metastable [OH(OH_2_)_3_]^−^ anion is generated by treatment of a strongly basic phosphazene (Schwesinger base) with four equivalents of water. Thermolysis under vacuum results in the decomposition of the hydroxide salt and quantitative liberation of the free phosphazene base. This process was used to synthesize the Schwesinger base on a larger scale.

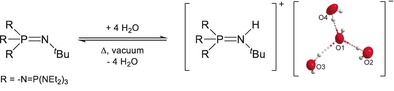

Oxonium and hydroxide ions play major roles in aqueous chemistry, and they serve as key subjects of numerous quantum‐chemical calculations. Thus, currently a considerable number of reports focus on oxonium ions of the general formula [(OH_3_)_*n*_(OH_2_)_*m*_)]^*n*+^ such as, for example, the “Zundel cation” [(OH_3_)(OH_2_)]^+^ 1 and the “Eigen cation” [(OH_3_)(OH_2_)_3_]^+^.^2^ Their existence is closely related to the presence of weakly coordinating anions.^3^

Considering the many variations of hydrated oxonium salts in the liquid or solid state, information on isolated hydrates of the hydroxide anion is rare.^4^ In contrast to the gas phase where different anions are evidenced by theoretical calculation, for example, the local mode calculations on MP2 level,^5^ isolated hydrated hydroxides have not been unambiguously documented.^5^, ^6^ In 1978 Raymond et al. reported the geometrical structure of the anion [OH(OH_2_)]^−^.^7^ This anion, however, is packed in a network of 18 water molecules and also has significant contacts to the sodium counterions. Clearly, such a species does not fulfil the requirements of an isolated hydrated OH^−^ anion. Thus, it is obvious that the presence of weakly Lewis acidic cations is a prerequisite for the observation of well‐separated hydroxide ions solvated by a shell of water molecules.

Phosphazene superbases, introduced by Schwesinger et al. in 1987,^8^, ^9^, ^10^ exhibit extremely high ^MeCN^ *p*K${}_{\mathrm{BH}{}^{+}}$ values, such as 26.9^11^ for monophosphazene **1** up to 42.7^9^ for tetraphosphazene **2** (Figure 1). The Schwesinger base **2** has found many applications in, for example, the anti‐Markovnikov addition of alcohols to aryl alkenes,^12^ Ullmann couplings,^13^ as well as ether deprotonation processes.^14^

**Figure 1:**
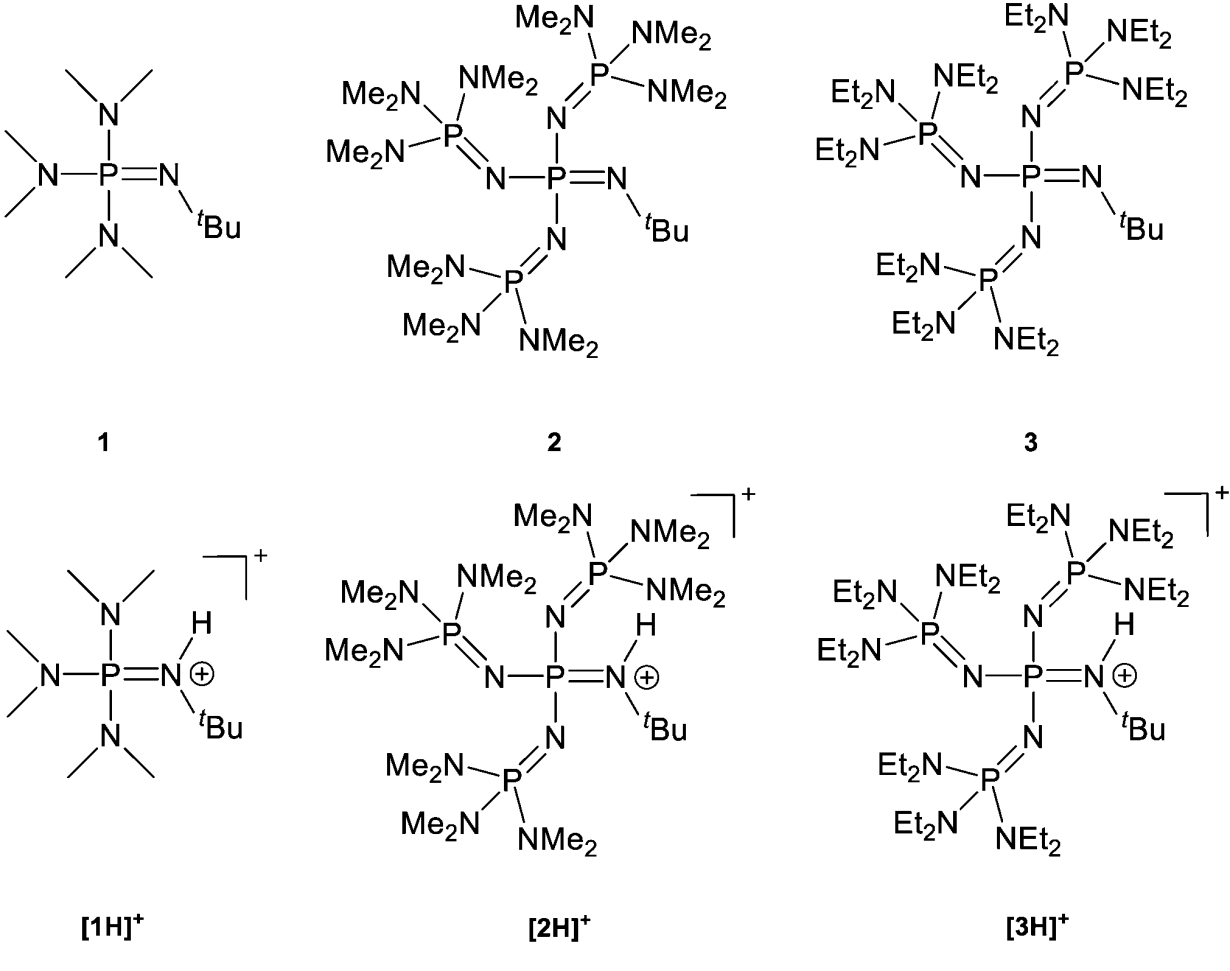
Overview of free and protonated mono‐ (**1**) and tetraphosphazene (**2**) bases, published by Schwesinger et al.,^10^ and the more weakly coordinating phosphazenium cation **[3H]^+^** of this work.

For the preparation of naked and highly reactive anions like the fluoride anion,^15^ phosphazenium counterions are particularly beneficial due to their low electrophilicity. For our investigation of hydroxide‐based water clusters, we envisaged ion separations as large as possible. For this purpose Schwesinger bases like **2** (Figure 1) seem promising. A compound having an increased volume should also have a lower tendency to add nucleophiles. Moreover, deprotonation processes at the iminium functionality of the corresponding acid should be considerably impeded. An obvious route to this objective should be the replacement of the dimethylamino substituents in **2** by bulkier diethylamino groups, as in **3**. The synthesis of this derivative was realized by a slight modification of the published procedure as depicted in Scheme 1.^8^, ^9^, ^10^

**Scheme 1:**
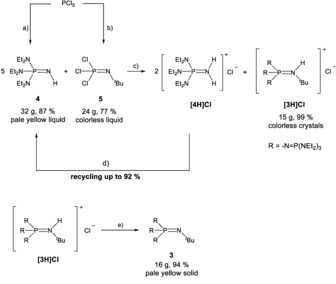
Synthesis of **3**. a) 1. HNEt_2_ (6 equiv.), CH_2_Cl_2_, −30 °C, −3 [H_2_NEt_2_]Cl; 2. NH_3_ (2 equiv.), CH_2_Cl_2_, −20 °C, −[NH_4_]Cl; 3. KO*t*Bu, MeOH, 0 °C, −KCl, −HO*t*Bu. b) H_2_N*t*Bu (3 equiv.), *n*‐pentane, −2 [H_3_N*t*Bu]Cl. c) 160 °C, 3 days. d) KO*t*Bu, MeOH, 0 °C, −KCl, −HO*t*Bu. e) NaNH_2_, NH_3_, −70 °C to rt, −NaCl, −NH_3_.

Compound Cl_3_PN*t*Bu (**5**) was prepared from phosphorus pentachloride and *tert*‐butylamine in a Kirsanov‐type reaction.^10^, ^11^, ^16^ Compound (Et_2_N)_3_PNH (**4**) is accessible in analogy to the synthesis of (Me_2_N)_3_PNH as previously devised by Schwesinger et al.^9^ Combination of neat **4** and **5** resulted in a nucleophilic substitution with the formation of phosphazenium chloride [{Et_2_N)_3_P=N}_3_P=NH(*t*Bu)]Cl (**[3H]Cl**) in high yield. In the ^31^P NMR spectrum of salt **[3H]Cl** the central phosphorus atom was observed as a quartet of doublets at *δ*=−33.9 ppm with coupling constants of ^2^ *J*_PP_=70 Hz and ^2^ *J*_PH_=8 Hz. The phosphorus atoms of the three peripheral phosphazenyl substituents gave rise to a signal at *δ*=7.4 ppm, which is split into a doublet of tridecets with a ^2^ *J*_PP_ coupling constant of 70 Hz and a ^3^ *J*_PH_ coupling constant of 10 Hz to the 12 methylene protons of the ethyl units.^17^

The crystal structure^18^ of **[3H]Cl** was elucidated by X‐ray crystallography. Suitable single crystals were grown from a saturated ethereal solution at −28 °C (Figure 2). The hydrogen atom bonded to N1 could be refined isotropically. The nitrogen atom N1 of the iminium group is slightly pyramidalized (sum of angles 350.6°). The bond length P1−N1 of 167.2(2) pm is significantly longer than the bonds P1−N2, P1−N3 and P1−N4, which range from 158.5(2) to 160.6(2) pm.

**Figure 2:**
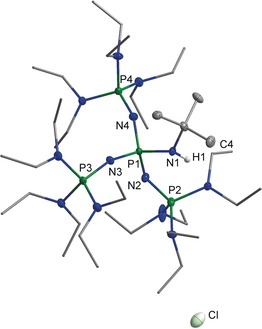
Molecular structure of the salt **[3H]Cl**. Thermal ellipsoids are shown at 50 % probability. Hydrogen atoms bonded at carbon and one minor occupied disordered ethyl group are omitted for clarity. Diethylamino groups are shown simplified as stick model. Selected bond lengths [pm] and angles [°]: P1−N1 167.2(2), P1−N2 160.6(2), P1−N3 158.5(2), P1−N4 158.9(2), P2−N2 155.4(2), N1−C1 148.0(3); C1−N1−P1 128.6(2), N1−P1−N4 109.0(1), P1−N2−P2 140.7(1).

The byproduct **[4H]Cl** can be isolated by aqueous extraction from the reaction mixture. Deprotonation results in the regeneration of **4** in high yields of about 92 %, which improves the waste‐to‐product ratio. Deprotonation of **[3H]Cl** to the free base **3** was achieved in 94 % yield by treatment with sodium amide in liquid ammonia (Scheme 1).^17^ The molecular structure^18^ of **3** was ascertained by X‐ray diffractometry utilizing single crystals grown in a saturated solution of the compound in *n*‐hexane at −28 °C (Figure 3). Phosphazene **3** crystallizes in the orthorhombic space group *Pbca*. In comparison to its corresponding acid, the atomic distance P1−N1 is shortened to 157.8(1) pm, representing a double bond. The bonds P1−N2, P1−N3, and P1−N4 range from 163.2(2) to 163.8(2) pm and also point to some degree of multiple bonding.^19^

**Figure 3:**
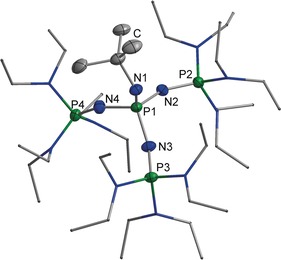
Molecular structure of **3**. Thermal ellipsoids are shown at 50 % probability. Hydrogen atoms are omitted for clarity. Diethylamino groups are shown simplified as a stick model. Selected bond lengths [pm] and angles [°]: P1−N1 157.8(1), P1−N2 163.8(2), P1−N3 163.1(1), P1−N4 163.2(2), P2−N2 153.0(1), N1−C1 145.7(2); C1−N1−P1 126.1(1), N1−P1−N4 113.1(1), P1−N2−P2 150.3(1).

For the generation of isolated hydroxide/water clusters, different quantities of water were added to solutions of phosphazene **3** in *n*‐hexane. The resulting phosphazenium hydroxides are soluble in chlorobenzene. At room temperature, however, such solutions slowly decompose. This product separates from polar solvents as an oil, whereas amorphous or crystalline samples are obtained from apolar solvents like *n*‐hexane. This finding may be rationalized by the various amounts of water incorporated in the precipitates, which hampered the isolation of a well‐defined bulk material. However, colorless crystals were obtained after slow evaporation of a methanol/water solution of **3** at room temperature and atmospheric pressure. The result of the elemental analysis revealed a possible hydroxide hexahydrate salt of the protonated phosphazene, [OH(OH_2_)_6_]^−^ (calcd: C 47.46, H 11.25, N 17.99, P 12.24, O 11.06; found: C 47.12, H 11.21, N 17.75, P 12.07, O 11.34). Since the crystals did not show any diffraction pattern, they could not be analyzed by X‐ray diffraction. Nevertheless, a single crystal of **[3H][OH(OH_2_)_3_]** was isolated by slow diffusion of water into an *n*‐hexane solution of the base.^17^ The X‐ray crystallographic analysis^18^ revealed a disorder of two ethyl groups of the cation with a ratio of 82:18 and a disorder of the anion with the same ratio (Figure 4 A). The hydrogen atoms of the protonated nitrogen atom N4 and the major occupied part of the disordered anion could be refined isotropically, the latter with fixed O−H distances and angles. Looking at the ions of the reliably modelled major part, the shortest separation between an oxygen atom and the cation can be measured for O4–C10′ (symmetry code 1−*x*,1−*y*,1−*z*) with 339.4(3) pm. This distance is clearly greater than the van der Waals radii and shows that this is the first example of an isolated hydroxide hydrate anion that is not in direct contact to a cation. The O−O distances are in the range of 251.6(1) pm to 260.2(3) pm. The calculated value of the *C*_3_‐symmetrical hydroxide shows a slightly longer distance of 261.2 pm (Figure 4 B).^5^ The experimental O−O−O angles range from 88.7(1)° to 115.9(1)° and include the calculated angle of 102.4°.

**Figure 4:**
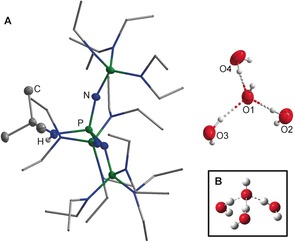
A) Molecular structure of **[3H][OH(OH_2_)_3_]**. Thermal ellipsoids are shown at 50 % probability. Hydrogen atoms and minor occupied disordered parts are omitted for clarity. Diethylamino groups are shown simplified as a stick model. The anion is disordered (82:18). The hydrogens of the disordered water molecules could not be located reliably. Disorder of two ethyl groups (C33, C34, C35, C36) over two sites (82:18). Selected bond lengths [pm] and angles [°]: O1−O2 256.0(3), O1−O3 251.6(3), O1−O4 260.2(3); O2−O1−O4 88.7(1), O3−O1−O4 115.9(1), O2−O1−O3 110.4(1). B) Calculated *C*_3_‐symmetrical hydroxide trihydrate (MP2/6‐311++G(3df,3pd)).^5^

Since the phosphazenium hydroxide **[3H][OH(OH_2_)_3_]** is highly sensitive towards loss of water under reduced pressure, reliable elemental analyses are nearly impossible to obtain (Scheme 2). An IR spectrum of the product displays a very broad band at 3411 cm^−1^ for the OH stretching vibration of the hydroxide anion and the bonded water molecules. No discrete bands are indicated, which points to a fast proton exchange between the water molecules and the hydroxide anion.

**Scheme 2:**
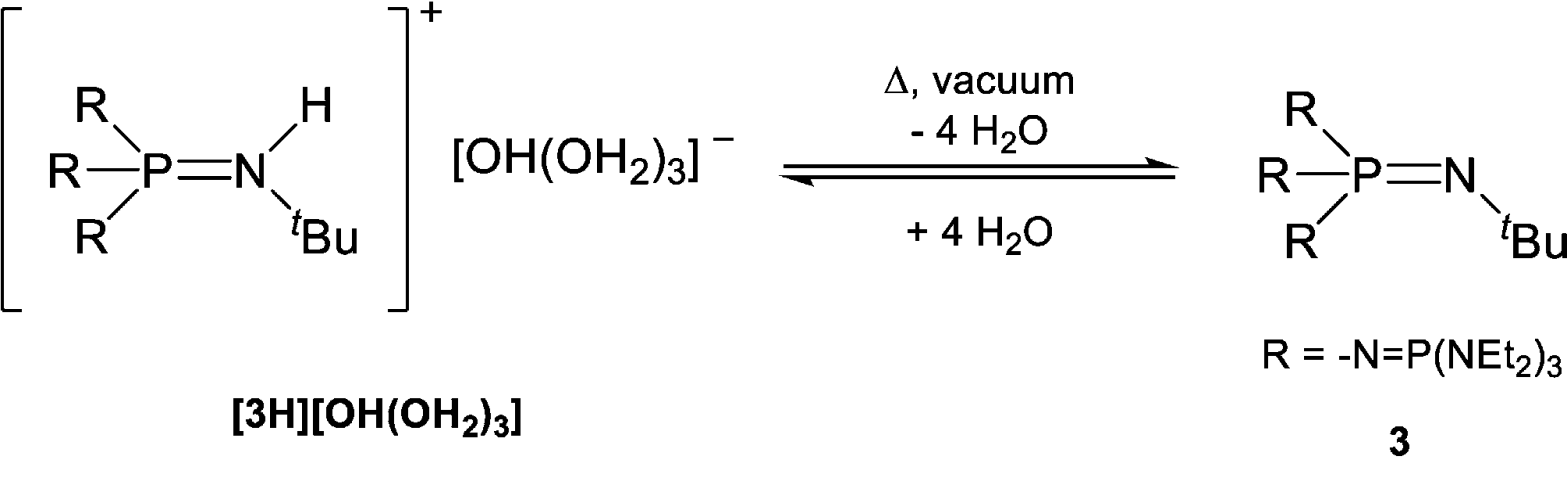
Equilibrium reaction of **3** and H_2_O.

To prove the existence of **[3H][OH(OH_2_)_3_]**, a chlorobenzene solution of **3** was titrated with water and analyzed by ^31^P NMR spectroscopy (Figure 5). Complete protonation of **3** is observed only when four equivalents of water or more are employed, and was evidenced by the characteristic ^2^ *J*_PP_ coupling constant of 70 Hz. The ^2^ *J*_PH_ coupling constant of 8 Hz for the protonated phosphazenium **[3H]^+^** could only be resolved when an excess of water was used. This phenomenon is probably due to a dynamic proton exchange (Scheme 2). Addition of less than four equivalents of water leads to an upfield shift of the signal for the central phosphorus atom from −31.3 ppm to −34.2 ppm with an increase in the ^2^ *J*_PP_ coupling constant from 29 Hz to 70 Hz (Figure 5). In the ^1^H NMR spectrum a broad signal at 4.9 ppm is observed for the protons of water and the hydroxide ion. The hydroxide hydrate decomposes at ambient temperature under vacuum, leading to the liberation of the free base **3**. Based on the collected data we suggest that the water molecules are activated or oriented in the superbasic system. The small size of a water molecule as well as the high basicity of the hydroxide anion render the deprotonation of the shielded iminium unit possible. In comparison to hydroxides with coordinating cations like alkali metal cations, a naked hydroxide anion in the presence of **[3H]^+^** seems to be unstable and therefore not preparable. The stabilizing effect of hydrogen‐bonding water molecules is necessary to lower the basicity of the hydroxide anion. This principle seems to be responsible for the selective generation of **3** from its hydroxide. Following the removal of stabilizing water molecules in vacuum, the basicity of the resulting anion is high enough for a selective deprotonation of the phosphazenium cation.

**Figure 5:**
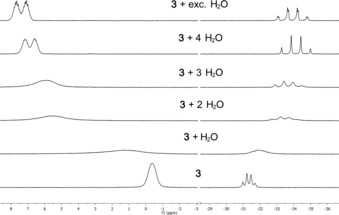
^31^P NMR spectroscopic titration of **3** with different amounts of water in chlorobenzene. Lock with [D_6_]acetone in a capillary.

The preparation of **3** by deprotonation of **[3H]Cl** with self‐igniting metal amides in liquid ammonia as a solvent is hazardous and problematic in view of waste disposal. Extensive cooling is expensive and upscaling to obtain base **3** in larger quantities remains a challenge. Reaction with potassium *tert*‐butanolate does not provide base **3** free of alcohol as claimed earlier by Schwesinger et al.^9^, ^10^ Less basic phosphazenes like **1**, indeed, could be liberated from the corresponding phosphazenium salts with the aid of potassium *tert*‐butanolate or even KOH, prior to distillation.^11^

A viable and elegant conversion of **[3H]Cl** into free **3** on a larger scale makes use of the intermediacy of reactive hydroxide hydrate **[3H][OH(OH_2_)_3_]**. Following a procedure by Taylor and Hupfield^20^ for the conversion of phosphazenium chlorides into their hydroxide by means of anion‐exchange resins, we succeeded in the clean formation of the hydroxide trihydrate **[3H][OH(OH_2_)_3_]** by exposing solutions of **[3H]Cl** to a strongly basic OH^−^ ion‐exchange resin (Scheme 3). The free, anhydrous base **3** was isolated after thermolysis of **[3H][OH(OH_2_)_3_]** in high vacuum at 70–100 °C (yield >97 %). Following a protocol disclosed in the literature, the anion‐exchange resin was regenerated with 1 m aqueous sodium hydroxide solution.^20^ In conclusion, our sequence represents a straightforward and selective route for the deprotonation of **[3H]Cl**.

**Scheme 3:**
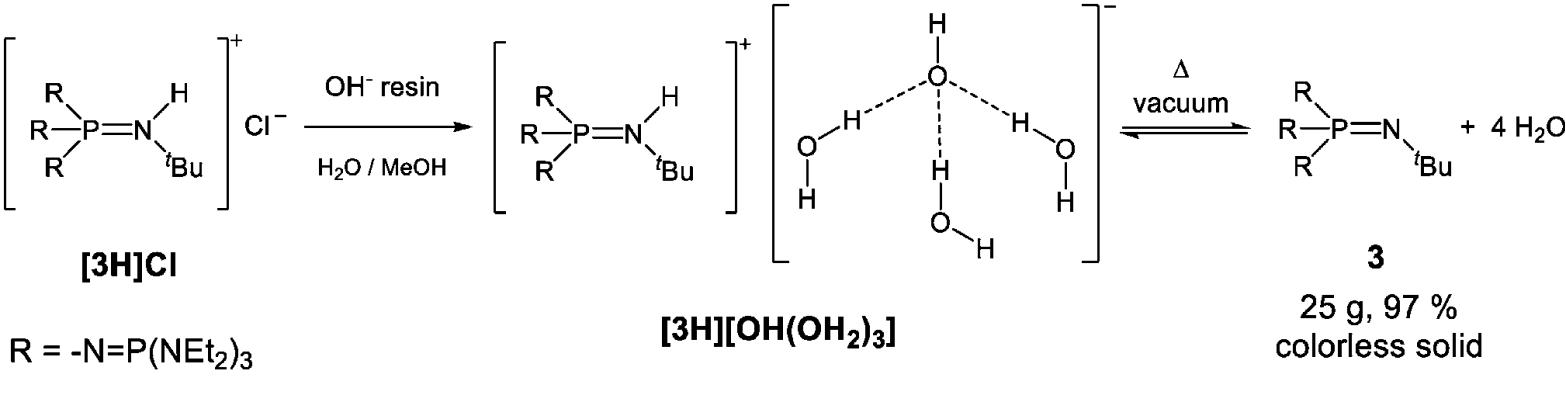
Synthesis of **3** using a salt‐exchange reaction to generate the metastable hydroxide hydrate **[3H][OH(OH_2_)_3_]**.

We used this method for the phosphazene‐base‐catalyzed trifluoromethylation reaction of Me_3_SiCl to generate the Ruppert–Prakash reagent, as shown in Scheme 4. The Ruppert–Prakash reagent Me_3_SiCF_3_ is the most commonly used trifluoromethylation agent in laboratory and industry,^21^, ^22^, ^23^ especially in medicinal chemistry for the preparation of trifluoromethyl‐substituted arenes, trifluoromethyl ethers, and trifluoromethyl ketones.^24^

**Scheme 4:**
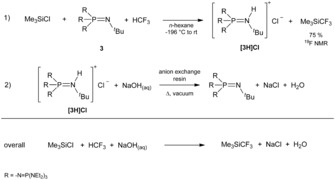
Overall reaction for the preparation of the Ruppert–Prakash reagent.

Fluoroform (HCF_3_) is an excellent and cheap source of the trifluoromethyl building block, since it is a waste product of Teflon production.^22^, ^25^ The deprotonation of HCF_3_ can be performed by using *n*‐butyllithium or potassium *tert*‐butanolate; however, the high fluorophilicity of alkali cations leads to a carbenoidic bond situation and rapid difluorocarbene elimination at low temperatures.^26^ Therefore the preparative trifluoromethylation reaction is accomplished via the detour of Me_3_SiCF_3._ Prakash et al. managed the synthesis of Me_3_SiCF_3_ in high yields from Me_3_SiCl, HCF_3_, and potassium bis(trimethylsilyl)amide (KHMDS) in toluene at low temperatures.^22^ Shibata et al. recently presented the possibility to lower the fluorophilicity of the potassium cation of trifluoromethyl potassium by the use of glyme as a coordinating solvent.^27^ Weakly coordinating phosphazenium cations also prevent the difluorocarbene elimination (Scheme 5) as presented by Shibata et al. and Zhang et al. for the trifluoromethylation of electrophilic carbonyl compounds^28^ and sulfonyl fluorides,^29^ as well as for different epoxides, carbon dioxide, and esters^30^ in good to excellent yields using **2** and fluoroform.

**Scheme 5:**
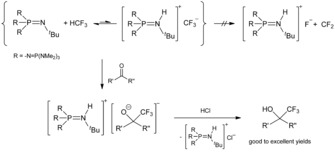
Trifluoromethylation of carbonyl compounds.^28^

The ^19^F and ^31^P NMR spectroscopic investigation of a mixture of **3** and fluoroform does not show any reaction, as reported by Zhang for the methyl derivative **2**.^30^ The addition of electrophiles leads to a rapid trifluoromethylation at ambient temperature (Scheme 5). Phosphazene **3** reacts rapidly with Me_3_SiCF_3_, forming Me_3_SiF and a wide range of byproducts due to a difluorocarbene elimination. The decomposition reaction of Me_3_SiCF_3_ with fluoride salts was already studied by Tyrra and Naumann et al. and resulted in perfluoroalkylated polyanions.^31^ By using an excess of Me_3_SiCl, the reaction of **3** and HCF_3_ can be controlled kinetically leading to the selective formation of Me_3_SiCF_3_ (−67.7 ppm in the ^19^F NMR spectrum) with yields of up to 75 % (Scheme 4).^17^

The use of a hydrocarbon solvent is crucial, because the high basicity of phosphazene **3** causes solvent deprotonation in the presence of Lewis acidic components like Me_3_SiCl. The high molecular weight and high cost of phosphazenes **2** and **3** make trifluoromethylation unprofitable on a larger scale. For this reason, Zhang and Shibata suggested the use of additives such as bis(trimethylsilyl)amine and use of **2** in catalytic amounts of about 20 mol %.^29^, ^30^ The use of additives leads to a loss of high amounts of the Schwesinger base during the conversion on multimolar scales. In our case the precipitated phosphazenium hydrochloride **[3H]Cl** in the trifluoromethylation reaction (Scheme 4) was regenerated in excellent yields of about 98 to 100 % after every reaction step through the use of an anion‐exchange resin, as shown in Table 1 in the Supporting Information,^17^ whereas small amounts of **3** are used for ^31^P NMR spectroscopic investigations after every step. Thus we showed the recovery and the possibility to use phosphazene **3** in further trifluoromethylation reactions without any loss of reactivity.

In the overall reaction for the synthesis of the Ruppert–Prakash reagent, as shown in Scheme 4, the trifluoromethylation reaction is accomplished by the use of sodium hydroxide as a base. The pentafluoroethylation of Me_3_SiCl with pentafluoroethane and **3** results in the formation of trimethylpentafluoroethylsilane in yields of about 61 %.^17^ In this reaction decomposition of **3** is not observed. This recommends the use of phosphazene **3** for further fluoro‐ and perfluoroalkylation reactions.

In conclusion, we have reported the first structurally characterized metastable hydroxide trihydrate **[OH(OH_2_)_3_]^−^**, generated via the newly prepared phosphazene base **3**. The hydroxide shows the tendency to lose water under vacuum, which effects selective deprotonation of the phosphazenium cation **[3H]^+^**. This protocol is used for the selective formation of **3** from its hydrochloride by means of a basic anion‐exchange resin in excellent yields of over 97 % and circumvents the use of hazardous metal amides in liquid ammonia which allows the production on a larger scale (>25 g). We also described the synthesis of the Ruppert–Prakash reagent Me_3_SiCF_3_ in yields of about 75 % by using fluoroform (HCF_3_), Me_3_SiCl, and Schwesinger base **3**. The free base **3** was regenerated by an anion‐exchange resin from precipitated phosphazenium chloride **[3H]Cl** in excellent yields of over 98 % and reused for the trifluoromethylation reaction without a loss of reactivity. Since the exchange resin can be regenerated with aqueous sodium hydroxide solution, in the overall reaction the trifluoromethylation is carried out using sodium hydroxide as base.

## Conflict of interest

The authors declare no conflict of interest.

## Supporting information

As a service to our authors and readers, this journal provides supporting information supplied by the authors. Such materials are peer reviewed and may be re‐organized for online delivery, but are not copy‐edited or typeset. Technical support issues arising from supporting information (other than missing files) should be addressed to the authors.

Supplementary
