# Supplementary material for: Generation and Applications of the Hydroxide Trihydrate Anion, [OH(OH2)3]−, Stabilized by a Weakly Coordinating Cation
Source: Angew Chem Int Ed Engl. 2019 Sep 9;58(41):14633–8. doi: 10.1002/anie.201908589 (PMC6790940; doi:10.1002/anie.201908589)
Supplement: Supplementary file 1 — Supplementary [file ANIE-58-14633-s001.pdf]

Angewandte Chemie
Eine Zeitschrift der Gesellschaft Deutscher Chemiker

Supporting Information

Generation and Applications of the Hydroxide Trihydrate Anion, $[OH(OH_2)_3]^-$, Stabilized by a Weakly Coordinating Cation

*Robin F. Weitkamp, Beate Neumann, Hans-Georg Stammler, and Berthold Hoge**

anie_201908589_sm_miscellaneous_information.pdf

1. Experimental Section

1.1 General Part

All chemicals were obtained from commercial sources and used without further purification. Standard high-vacuum techniques were employed throughout all preparative procedures, except aqueous workups. Non-volatile compounds were handled in a dry N_2 atmosphere using Schlenk techniques.

1.2 Analysis Methods

1.2.1 NMR Spectroscopy

NMR spectra were recorded on a Bruker Model Avance III 300 spectrometer (^{1}H 300.13 MHz; ^{13}C 75.47 MHz; ^{19}F 282.40 MHz; ^{31}P 121.49 MHz). Positive shifts are downfield from the external standards TMS (^{1}H, ^{13}C), CCl_3F (^{19}F) and H_3PO_4 (^{31}P). The NMR spectra were recorded in the indicated deuterated solvent or in relation to acetone-d_6-filled capillaries.

1.2.2 IR Spectroscopy

IR spectra were recorded on an ALPHA-FT-IR spectrometer (Bruker) using an ATR unit with a diamond crystal for liquids and solids.

1.2.3 Elemental Analyses

Elemental analyses were performed by Mikroanalytisches Laboratorium Kolbe (Oberhausen, Germany).

1.2.4 Melting Point

Melting points were measured on a Mettler Toledo Mp70 Melting Point System.

1.3 Syntheses

1.3.1 Synthesis of $(Et_2N)_3PNH$ (4)

Phosphorus pentachloride (29.62 g, 142.2 mmol) is suspended in 250 mL of dichloromethane at -78 °C. A solution of diethylamine (6.6 eq, 98.0 mL, 944 mmol) in 50 mL of dichloromethane is added dropwise, so that the inner temperature is kept below -30 °C. After the addition, the orange slurry is allowed to warm to room temperature. After an additional hour of stirring, the mixture is cooled to -20 °C and gaseous ammonia is bubbled into the reaction mixture via a syringe over a period of 50 minutes. After positive ^{31}P NMR control, the suspension is filtrated over a Schlenk frit and the colorless solid is washed with dichloromethane (3 x 25 mL). The solvent is removed under reduced pressure, leading to a hygroscopic orange solid (60.78 g), containing diethylammonium chloride. The product is dissolved in 60 mL of methanol at -20 °C and a solution of potassium-*tert*-butanolate (65.85 g, 586.9 mmol) in 150 mL of methanol is added dropwise to the orange solution. After addition, the mixture is allowed to warm to room temperature and stirred overnight. The precipitate is filtered off and washed with methanol (3 x 20 mL). The filtrate is freed from solvent under reduced pressure. All volatile components are condensed into a second Schlenk flask (110 °C, 10^{-3} mbar). The yellow solution is distilled in vacuo, leading to a pale yellow liquid (32.39 g, 123.5 mmol, 87 % based on PCl_5, 10^{-3} mbar, 60-82 °C).

^{1}H NMR ($CDCl_3$, rt): δ [ppm] = 1.1 (t, d, $^{3}J_{HH}$ = 7 Hz, $^{4}J_{PH}$ = 1 Hz, 18 H, CH_3), 3.0 (d, q, $^{3}J_{PH}$ = 10 Hz, $^{3}J_{HH}$ = 7 Hz, 12 H, CH_2).
^{31}P NMR ($CDCl_3$, rt): δ [ppm] = 42.7 (tridec, $^{3}J_{PH}$ = 10 Hz).
IR (ATR): v [cm^{-1}] = 3403 (vw), 2967 (w), 2930 (w), 2867 (w), 1460 (w), 1376 (m), 1348 (w), 1296 (vw), 1201 (m), 1184 (s), 1096 (m), 1057 (w), 1015 (vs), 933 (s), 837 (w), 790 (m), 692 (s), 630 (w), 531 (w, br).
elemental analysis for $C_{12}H_{31}N_4P$ (262.4 g/mol): calcd.: C 54.93, H 11.91, N 21.35, P 11.81; found: C 54.61, H 11.91, N 21.48, P 11.68.

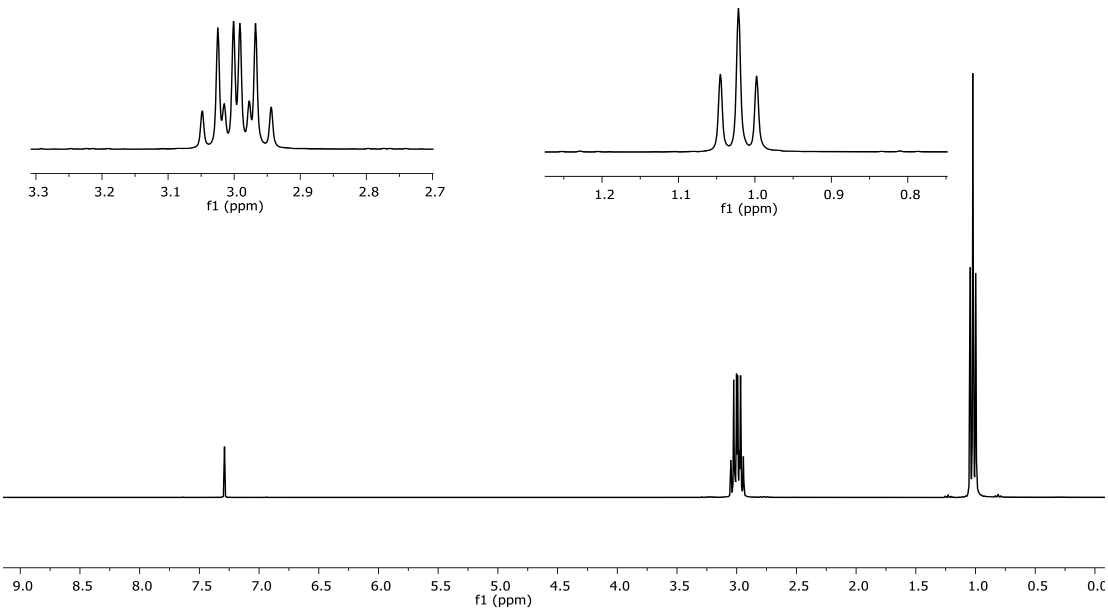

Figure 1. ^{1}H NMR spectrum of **4** in chloroform-d_1.

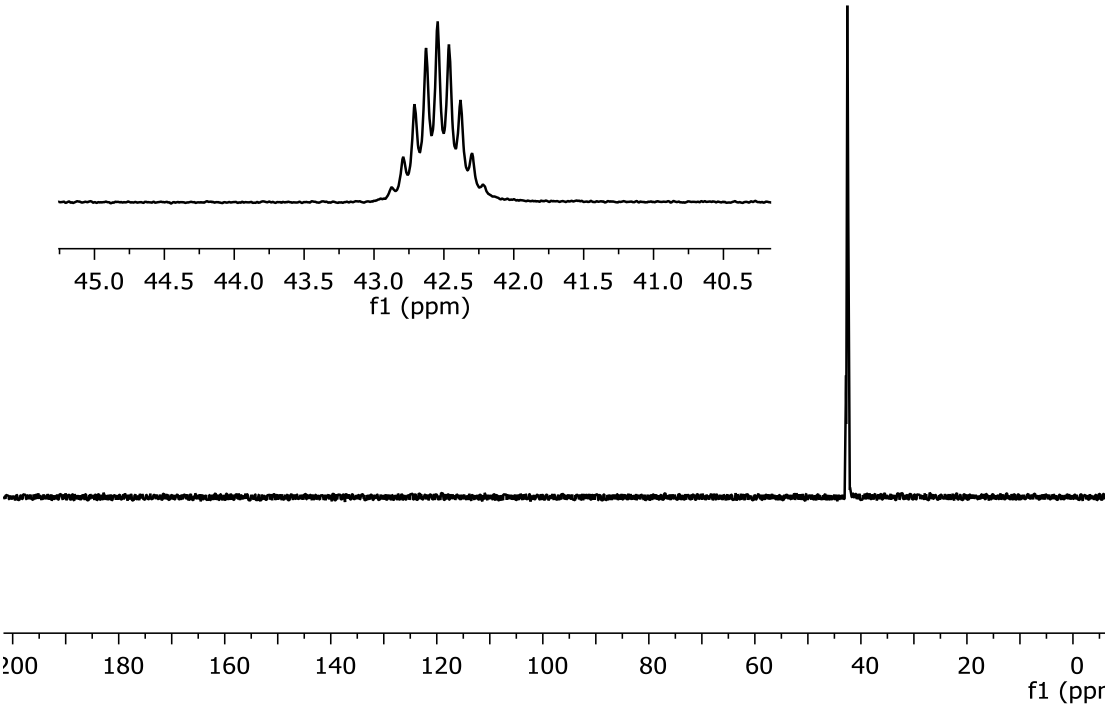

Figure 2. ^{31}P NMR spectrum of **4** in chloroform-d_1.

1.3.2 Synthesis of Cl_3PN*t*Bu (5)

Phosphorus pentachloride (31.53 g, 151.5 mmol) is suspended in 750 mL of *n*-pentane before a solution of *tert*-butylamine (3.1 eq, 34.34 g, 469.5 mmol) in 150 mL of *n*-pentane is added dropwise under ice bath cooling. Thereafter the suspension is refluxed for 1.5 h and then stirred overnight at room temperature. The precipitate is filtered off and washed with *n*-pentane (3 x 50 mL). After removing the solvent under reduced pressure, the colorless liquid residue is distilled in vacuo (25 mbar, 55 °C) leading to the product (24.21 g, 116.1 mmol, 77 % based on PCl_5) as a colorless liquid.

^{1}H NMR ($CDCl_3$, rt): δ [ppm] = 1.4 (d, $^4J_{PH}$ = 3 Hz).
^{31}P NMR ($CDCl_3$, rt): δ [ppm] = -78.3 (s).

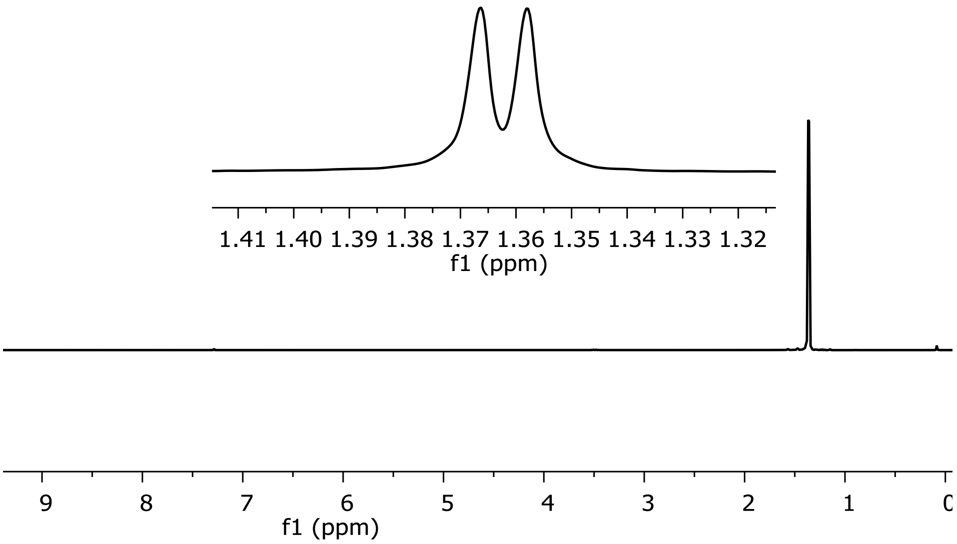

Figure 3. ^{1}H NMR spectrum of **5** in chloroform-d_1.

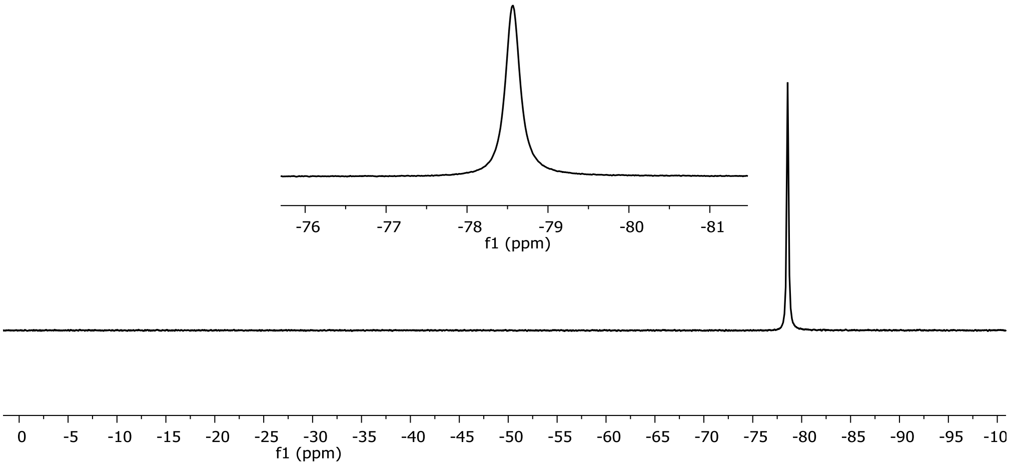

Figure 4. ^{31}P NMR spectrum of **5** in chloroform-d_1.

1.3.3 Synthesis of [{($Et_2N)_3PN\}_3PN(H)$*t*Bu]Cl ([3H]Cl)

At 0 °C a sample of **5** (3.40 g, 16.27 mmol) is added dropwise with a syringe to neat **4** (6 eq, 25.62 g, 97.64 mmol). The suspension is heated for three days at 160 °C in an evacuated Young-flask and afterwards extracted with boiling water (2 x 75 mL). The residue is dried in high vacuum prior to recrystallization from diethyl ether (30 mL) at -28 °C. The product (14.92 g, 16.17 mmol, 99 % based on Cl_3PN*t*Bu) is collected as a colorless solid (m.p. > 185 °C (dec.)).

^{1}H NMR ($CDCl_3$, rt): δ [ppm] = 1.1 (t, $^3J_{HH}$ = 7 Hz, 54 H, CH_3), 1.3 (s, 9 H, $C(CH_3)_3$), 2.0 (d, $^2J_{PH}$ = 8 Hz, 1 H, NH), 3.1 (d,q, $^3J_{PH}$ = 10 Hz, $^3J_{HH}$ = 7 Hz, 36 H, CH_2).
^{13}C{^{1}H}APT NMR ($CDCl_3$, rt): δ [ppm] = 13.5 (d, $^3J_{PC}$ = 4 Hz, CH_3), 31.4 (d, $^3J_{PC}$ = 5 Hz, C(**C**$H_3)_3$), 39.0 (d, $^2J_{PC}$ = 6 Hz, CH_2), 50.6 (d, $^2J_{PC}$ = 4 Hz, **C**$(CH_3)_3$).
^{31}P NMR ($CDCl_3$, rt): δ [ppm] = -33.9 (q, d, $^2J_{PP}$ = 70 Hz, $^2J_{PH}$ = 8 Hz, 1 P, P=NH), 7.4 (d, tridec, $^2J_{PP}$ = 70 Hz, $^3J_{PH}$ = 10 Hz, 3 P, $(Et_2N)_3P$).
IR (ATR): v [cm^{-1}] = 3387 (vw, vbr), 2967 (vw), 2931 (vw), 2870 (vw), 1633 (vw, br), 1463 (vw), 1379 (w), 1350 (w), 1265 (m, br), 1201 (m), 1173 (vs), 1107 (vw), 1054 (vw), 1016 (vs), 941 (s), 845 (vw), 792 (m), 740 (vw), 700 (m), 613 (w), 507 (s), 439 (m).
elemental analysis for $C_{40}H_{100}N_{13}P_4Cl$ (922.7 g/mol): calcd.: C 52.07, H 10.92, N 19.74, P 13.43; found: C 51.31, H 10.78, N 19.67, P 13.13.

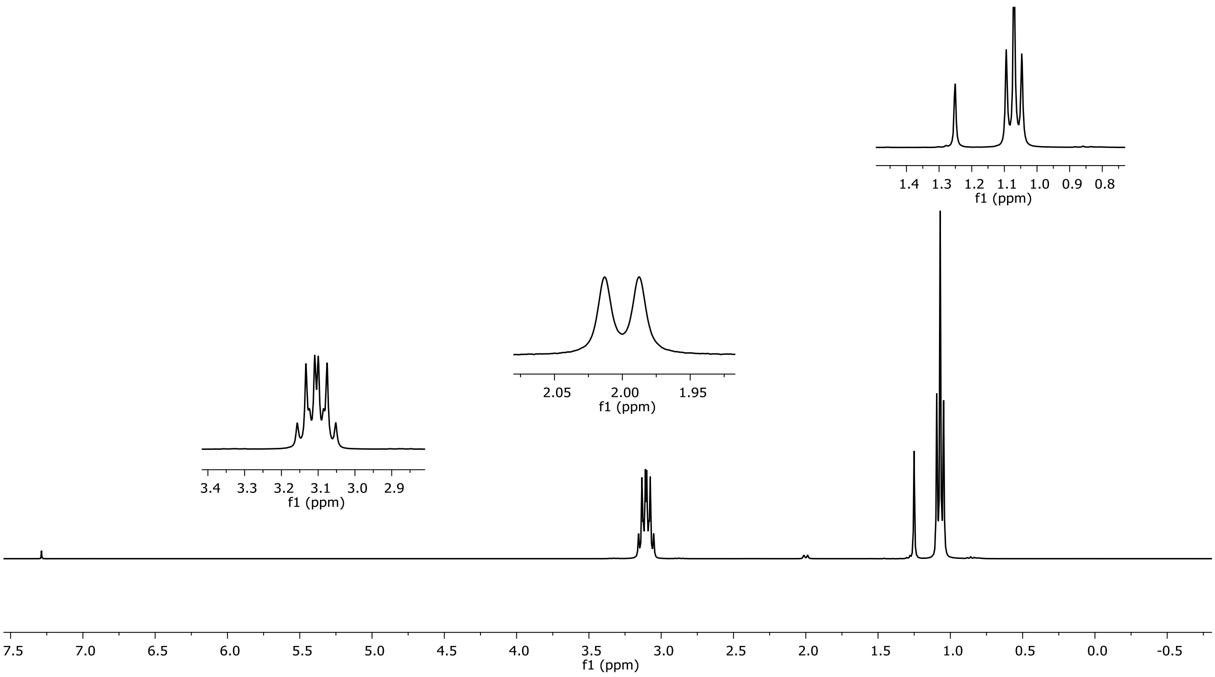

Figure 5. ^{1}H NMR spectrum of **[3H]Cl** in chloroform-d$_1$.

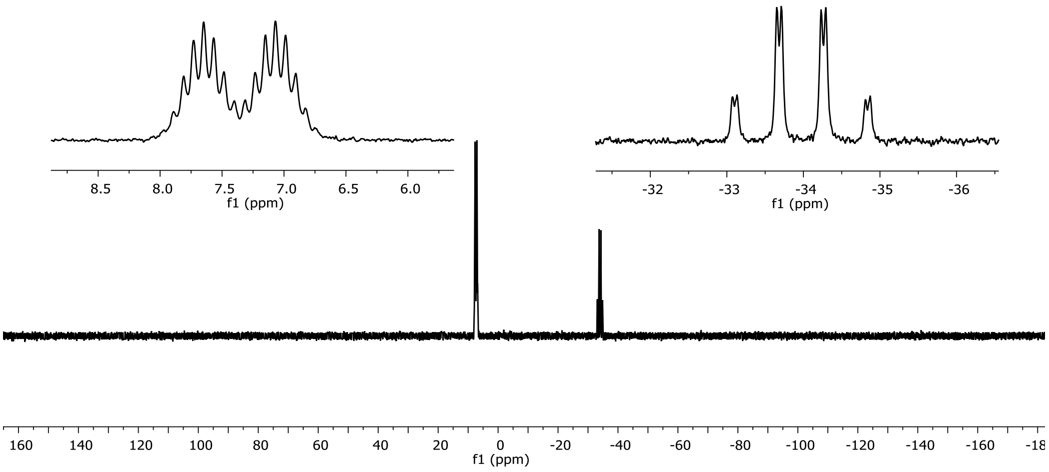

Figure 6. ^{31}P NMR spectrum of **[3H]Cl** in chloroform-d$_1$.

1.3.4 Synthesis of [$(Et_2N)_3PN]_3PN$*t*Bu (3)

a) Deprotonation via anion exchange resin

Hydrochloride **[3H]Cl** (26.33 g, 28.53 mmol) is dissolved in 60 mL of a methanol / water mixture (7 : 3) and passed three times through a strongly basic anion exchange resin (110 mL resin in 110 mL of MeOH / H_2O, Ion Exchanger III, 0.9 mol/L, Merck KGaA). The column is washed two times with 30 mL of a methanol / water mixture (7 : 3). The combined colorless solutions are evaporated in vacuo (10^{-3} mbar) at room temperature and the resulting solid is dried at 70 °C until no further solvent signal in the ^{1}H NMR spectrum is detectable. The product (24.55 g, 27.70 mmol, 97 %) is isolated as a bright yellowish solid (m.p. 230-240 °C (dec.)).

^{1}H NMR (C_6D_6, rt): δ [ppm] = 1.1 (t, $^3J_{HH}$ = 7 Hz, 54 H, CH_3), 1.7 (s, 9 H, $C(CH_3)_3$), 3.3 (d,q, $^3J_{PH}$ = 9 Hz, $^3J_{HH}$ = 7 Hz, 36 H, CH_2).
$^{13}C\{^1H\}$ NMR (C_6D_6, rt): δ [ppm] = 13.9 (d, $^3J_{PC}$ = 4 Hz, CH_3), 35.2 (d, $^3J_{PC}$ = 15 Hz, C(**C**$H_3)_3$), 39.4 (d, $^2J_{PC}$ = 5 Hz, CH_2), 51.0 (d, $^2J_{PC}$ = 5 Hz, **C**$(CH_3)_3$).
^{31}P NMR (C_6D_6, rt): δ [ppm] = -31.3 (q, $^2J_{PP}$ = 29 Hz, 1 P, P=N), 0.3 (m, 3 P, $(Et_2N)_3P$).
IR (ATR): ν [cm^{-1}] = 2968 (w), 2930 (vw), 2865 (w), 1462 (vw, br), 1374 (w), 1349 (w), 1267 (m), 1229 (m), 1181 (vs), 1104 (w), 1054 (w), 1015 (vs), 932 (s), 838 (w), 782 (m), 752 (w), 729 (w), 694 (s), 609 (w), 505 (s, br).
elemental analysis for $C_{40}H_{99}N_{13}P_4$ (886.2 g/mol): calcd.: C 54.21, H 11.26, N 20.55, P 13.98; found: C 53.31, H 11.07, N 20.21, P 14.34.

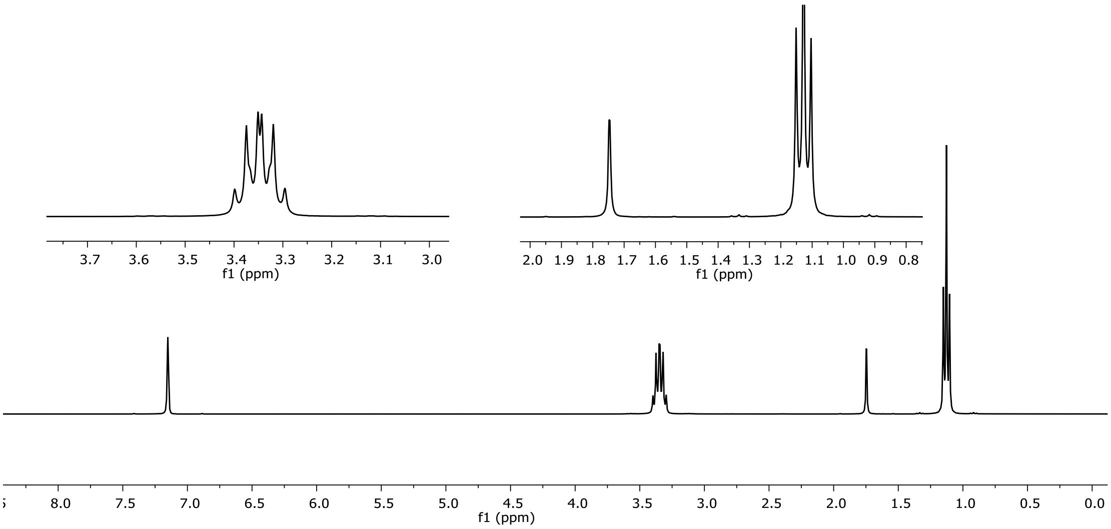

Figure 7. ^{1}H NMR spectrum of **3** in benzene-d_6.

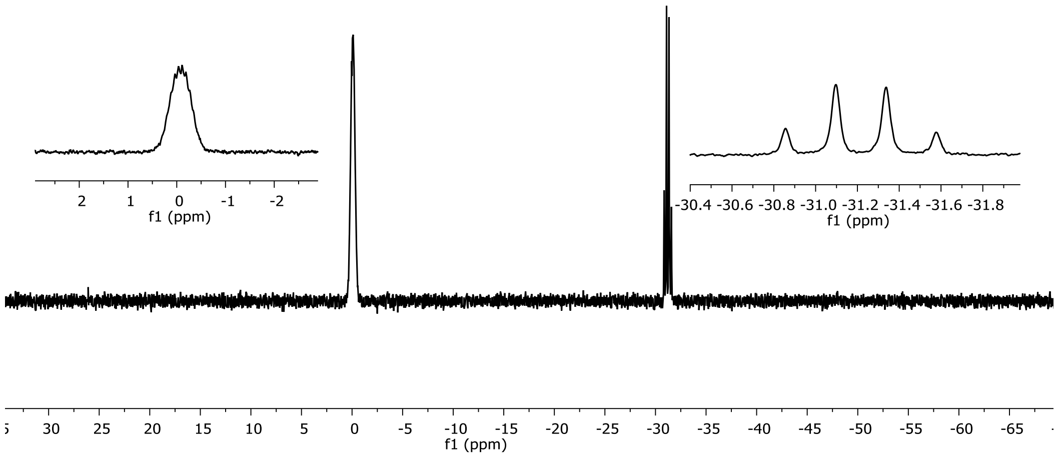

Figure 8. ^{31}P NMR spectrum of **3** in benzene-d$_6$.

b) Deprotonation via sodium amide

Ammonia (120 mL) is condensed onto sodium metal (1.13 g, 49.2 mmol) at -196 °C. In a cooling bath the suspension is allowed to warm to -70 °C and then a catalytic amount of iron(III)nitrate nonahydrate is added. The blue suspension is stirred for 2.5 h, until the suspension changed color from deep blue to brown. Then **[3H]Cl** (18.45 g, 20.0 mmol) is added and the suspension is allowed to warm to room temperature in the cooling bath overnight. The solid is suspended in 60 mL of *n*-hexane and the slurry is filtrated over a Schlenk-frit (P4). The residue is washed two times with 10 mL of *n*-hexane. The solvent is removed from the combined filtrates to give **3** (16.19 g, 18.7 mmol, 94 %) as a bright yellowish solid.

1.3.5 Generation of [{($Et_2N)_3PN\}_3PN(H)$*t*Bu][$OH(OH_2)_3$] ([3H][$OH(OH_2)_3$])

a) Generation in chlorobenzene solution

To a solution of **3** (556 mg, 0.63 mmol) in 4 mL of chlorobenzene, water (4 eq, 45 mg, 2.50 mmol) is added. ^{31}P NMR spectroscopic analysis shows complete protonation.

^{1}H NMR (C_6H_5Cl, rt): δ [ppm] = 0.7 (t, $^{3}J_{HH}$ = 7 Hz, 54 H, CH_3), 1.0 (s, 9 H, $C(CH_3)_3$), 2.7 (m, 36 H, CH_2), 4.9 (s, 8 H, OH).

^{31}P NMR (C_6H_5Cl, rt): δ [ppm] = -34.2 (q, $^{2}J_{PP}$ = 70 Hz, 1 P, P=NH), 6.9 (d, tridec, $^{2}J_{PP}$ = 70 Hz, $^{2}J_{PH}$ = 8 Hz, 3 P, $(Et_2N)_3P$).

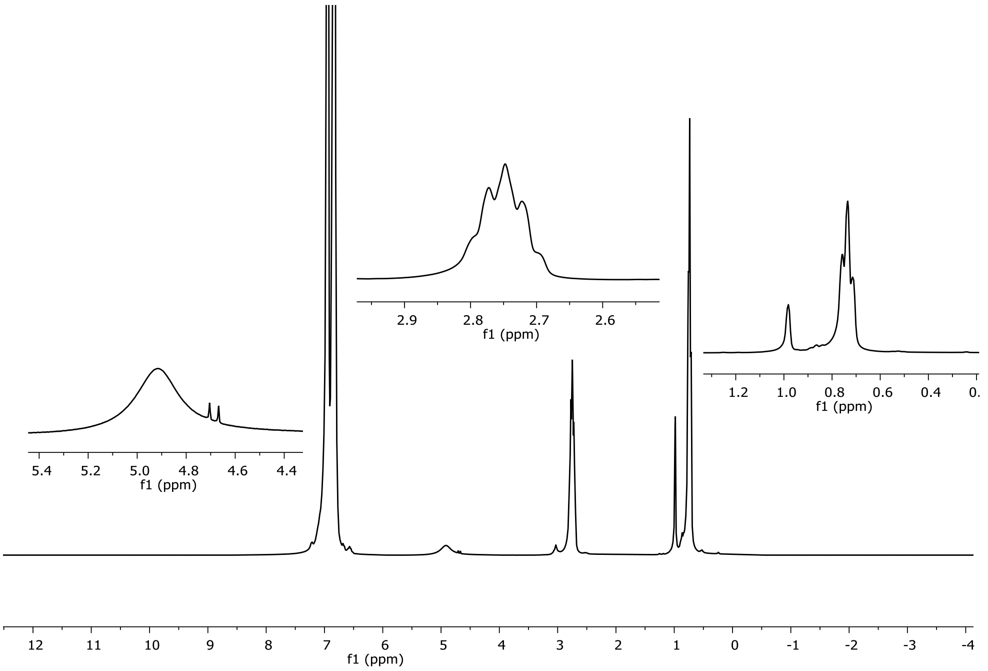

Figure 10. ^{1}H NMR spectrum of **[3H][$OH(OH_2)_3$]** generated in chlorobenzene (lock with acetone-d_6 in a capillary).

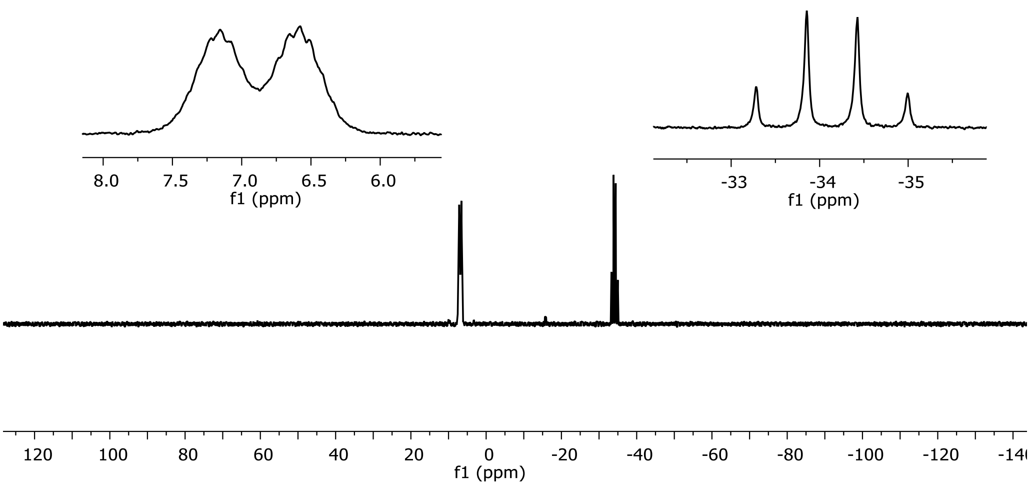

Figure 10. ^{31}P NMR spectrum of **[3H][OH(OH$_2$)$_3$]** generated in chlorobenzene (lock with acetone-d$_6$ in a capillary).

b) Generation from *n*-hexane solution

By slow diffusion of water into a solution of **3** (240 mg) in 4 mL of *n*-hexane a colorless solid is obtained.

IR (ATR): *v* [cm^{-1}] = 3411 (vw, vbr), 2969 (w), 2932 (w), 2871 (w), 1653 (w, br), 1464 (w), 1411 (w), 1379 (m), 1351 (w), 1271 (s, br), 1227 (w), 1202 (s), 1174 (vs), 1107 (w), 1055 (w), 1017 (vs), 942 (s), 921 (w), 845 (w), 792 (m), 740 (w), 699 (s), 612 (m), 507 (vs), 439 (s).

1.3.6 Synthesis of Me_3SiCF_3 and exemplary regeneration of 3

In a flame-dried flask equipped with a Young valve a sample of **3** (5.39 g, 6.08 mmol) is dissolved in 20 mL of *n*-hexane. The solution is degassed three times. Fluoroform (30 mmol) is condensed onto the solution at -196 °C and the mixture is allowed to melt for mixing. Me_3SiCl (7.11 mmol, 772 mg) is condensed onto the solution at -196 °C and the obtained emulsion is warmed to room temperature over a period of two hours. All volatile compounds are condensed into a second flask. The yield of Me_3SiCF_3 (69 %) is determined using ^{19}F NMR spectroscopy by adding 1,3-bis(trifluoromethyl)benzene (247 mg, 1.15 mmol). The precipitated solid **[3H]Cl** is dissolved in 20 mL of a methanol / water mixture (7 : 3) and passed three times through a strongly basic anion exchange resin (50 mL resin in 50 mL of MeOH / H_2O, Ion Exchanger III, 0.9 mol/L, Merck KGaA), after every run, the column is washed with additional 10 mL of MeOH / H_2O (7 : 3). The solvent of the combined fractions is removed under reduced pressure. The resulting solid residue is dried in high vacuum at 70 to 100 °C. Phosphazene **3** (5.30 g, 5.98 mmol, 98 %) is regenerated as a colorless solid. The purity is confirmed by ^{1}H and ^{31}P NMR spectroscopy (C_6D_6 as a solvent).

^{19}F NMR (*n*-hexane, rt): δ [ppm] = -67.7 (s, $^{1}J_{CF}$ = 320 Hz, $^{2}J_{SiF}$ = 40 Hz, CF_3).

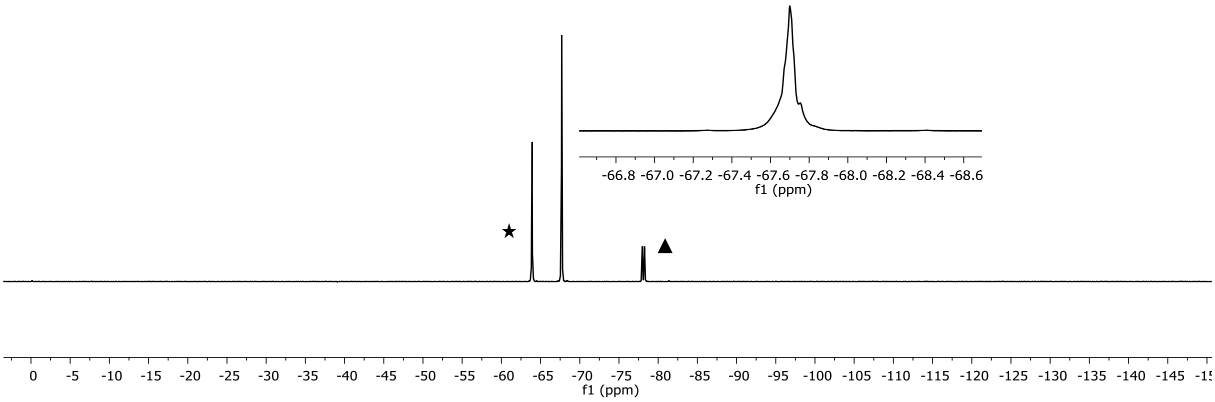

Figure 10. ^{19}F NMR spectrum of Me_3SiCF_3 generated in *n*-hexane (lock with acetone-d_6 in a capillary, 1,3-bis(trifluoromethyl)benzene as a standard). ★ 1,3-bis(trifluoromethyl)benzene, ▲ HCF_3.

Table 1. Regeneration of **3** with basic anion exchange resin.

Reaction[a]	**3**[b]	Me_3SiCF_3[c]	Recovery of **3**
1	6.08 mmol	69 %	98 %
2	5.79 mmol	74 %	98 %
3	5.57 mmol	75 %	100 %

[a] *n*-hexane, -196 °C to rt, 2 h. [b] Used quantity of **3**. [c] Yield determined via ^{19}F NMR spectroscopy using 1,3-bis(trifluoromethyl)benzene as standard, lock with acetone-d_6 in a capillary.

1.3.7 Synthesis of $Me_3SiC_2F_5$

In a flame-dried flask equipped with a Young valve a sample of **3** (4.84 g, 5.46 mmol) is dissolved in 25 mL of *n*-hexane and the solution is degassed three times. Pentafluoroethane (27.3 mmol) is condensed onto the solution at -196 °C and the mixture is allowed to melt for mixing. Me_3SiCl (5.82 mmol, 632 mg) is condensed onto the solution at -196 °C and the formed emulsion is allowed to warm to room temperature over a period of two hours. All volatile compounds are condensed into a second flask. The yield of $Me_3SiC_2F_5$ (61 %) is determined using ^{19}F NMR spectroscopy by adding 1,3-bis(trifluoromethyl)benzene (176 mg, 0.82 mmol).

^{19}F NMR (*n*-hexane, rt): δ [ppm] = -131.9 (s, $^1J_{CF}$ = 271 Hz, $^2J_{SiF}$ = 26 Hz, CF_2), -82.4 (s, $^1J_{CF}$ = 284 Hz, $^3J_{SiF}$ = 40 Hz, CF_3).

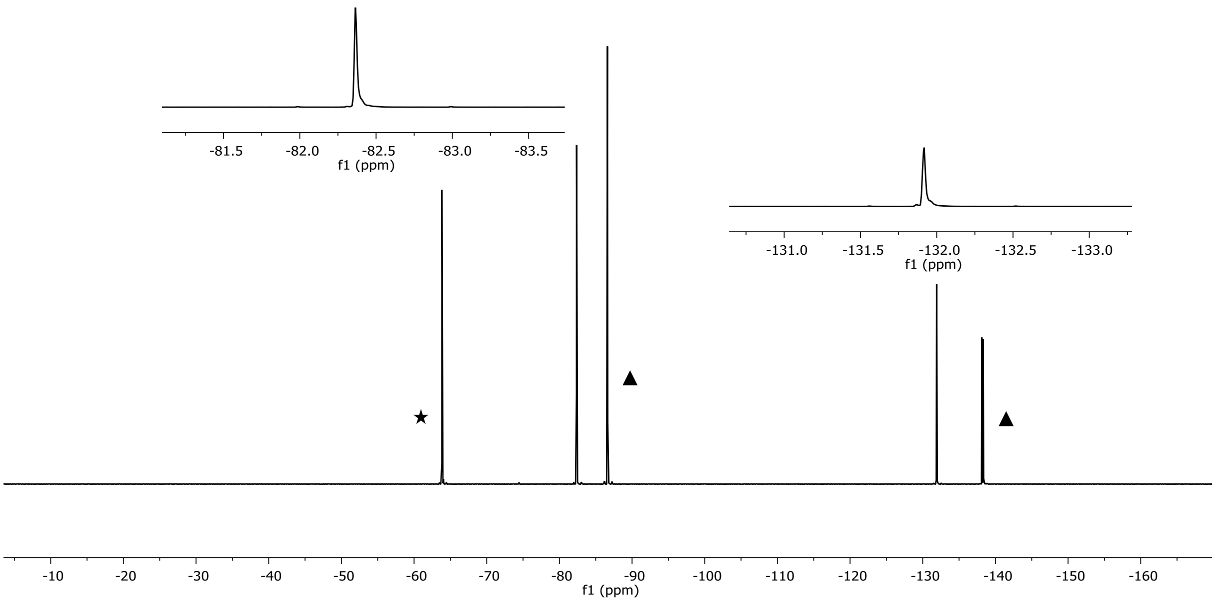

Figure 11. ^{19}F NMR spectrum of $Me_3SiC_2F_5$ generated in *n*-hexane (lock with acetone-d_6 in a capillary, 1,3-bis(trifluoromethyl)benzene as a standard). ★ 1,3-bis(trifluoromethyl)benzene, ▲ HC_2F_5.

1.4 Details on the X-Ray Diffraction

The crystal data were collected on a Rigaku Supernova diffractometer using graphite-monochromated Mo-K_α radiation (λ = 71.073 pm) or Cu-K_α radiation (λ = 154.184 pm) at 100.0(2) K.

Using Olex2[2], the structures were solved with the ShelXS[3] structure solution program using direct methods and refined with the ShelXL[4] refinement package using least squares minimization.

[3H]Cl showed a disorder of one ethyl group (C11/C12) over two sites (55:45)

[3H][OH(OH$_2$)$_3$] showed a disorder of two ethyl-groups (C33, C34, C35, C36) over two sites (82:18). The anion is disordered with the same ratio. N4 is protonated. All hydrogen atoms bonded at nitrogen or oxygen were refined isotropically, the ones in the major occupied part of the disordered anion with fixed O-H distances and H…H distances inside the water molecules. The hydrogen atoms of the minor part were neglected, but they were included in the sum formula for further calculations. An additional 1:1 disorder of one oxygen atom of the minor occupied part was modelled having in mind that the complete minor occupied part of the anion is most unreliable. The positions of the hydrogen atoms of the major occupied disorder of the anion can not be refined freely because some of the positions are disturbed by electron density of the minor occupied part. The small Uiso value of the hydrogen atom of the hydroxide reflects the high electron density inside this anion. Because of the separating of the hydroxide trihydrate anions by the weak coordinating cation only the half of the hydrogen atoms of the solvent water molecules are involved in hydrogen bonds. Details of the X-ray investigation are given in Table 2. CCDC 1938109 – 1938111 contain the supplementary crystallographic data for this paper. These data can be obtained free of charge via http://www.ccdc.cam.ac.uk/conts/retrieving.html.

Table 2. Structure refinement data of **[3H]Cl**, **3** and **[3H][OH(OH$_2$)$_3$].**

compound	**[3H]Cl**	**3**	**[3H][OH(OH$_2$)$_3$]**
Crystallographic Section			
empirical formula	$C_{40}H_{100}ClN_{13}P_4$	$C_{40}H_{99}N_{13}P_4$	$C_{40}H_{107}N_{13}O_4P_4$
a / pm	1260.86(2)	2231.95(4)	1103.99(3)
b / pm	2370.47(5)	1930.14(3)	2582.00(7)
c / pm	1767.13(3)	2437.55(4)	1969.22(7)
β	98.4343(17)	90	98.631(3)
V / 10^6 pm^3	5224.54(16)	10500.9(3)	5549.7(3)
Z	4	8	4
ρ_{calc} / mg·mm^{-3}	1.173	1.121	1.147
crystal system	Monoclinic	orthorhombic	monoclinic
space group	*Cc*	*Pbca*	$P2_1/n$
color shape	Colorless prisms	Colorless block	Colorless block
crystal size / mm^{-3}	0.23 × 0.14 × 0.05	0.37 × 0.18 × 0.06	0.44 × 0.19 × 0.13
Data collection			
μ / mm^{-1}	0.237	1.633	0.184
F(000)	2032.0	3920.0	2120
2θ range for data col. / °	3.4 to 60.1°	7.1 to 144.3°	3.2 to 60.3
index ranges	$-17 \le h \le 17$<br>$-33 \le k \le 33$<br>$-24 \le l \le 24$	$-27 \le h \le 25$<br>$-23 \le k \le 16$<br>$-30 \le l \le 29$	$-14 \le h \le 15$<br>$-28 \le k \le 35$<br>$-26 \le l \le 26$
reflections col.	37747	30476	51246
independent refl.	14863	10324	14683
R(int)	0.0376	0.0282	0.0367
data/restraints/ parameter	14863/2/568	10324/0/535	14683/7/637
goodness-of-fit on F^2	1.025	1.034	1.061
R_1 / wR_2 [I>2σ(I)]	0.0384/0.0802	0.0435/0.1170	0.0465/0.1093
R_1 / wR_2 (all data)	0.0458/0.0834	0.0494/0.1226	0.0676/0.1205
$\Delta\rho_{max/min}$ / e Å^{-3}	0.38/-0.36	0.78/-0.36	0.63/-0.45
Flack parameter	0.02(2)	n/a	n/a
CCDC number	1938109	1938110	1938111

2 References

[1] Matthew Monroe, *Molecular Weight Calculator*, **2012**.

[2] O. V. Dolomanov, L. J. Bourhis, R. J. Gildea, J. A. K. Howard, H. Puschmann, *J. Appl. Cryst.* **2009**, *42*, 339.

[3] G. M. Sheldrick, *Acta Cryst. A* **2015**, *71*, 3.

[4] G. M. Sheldrick, *Acta Cryst. C* **2015**, *71*, 3.
